# Exploring moral algorithm preferences in autonomous vehicle dilemmas: an empirical study

**DOI:** 10.3389/fpsyg.2023.1229245

**Published:** 2023-11-28

**Authors:** Tingting Sui

**Affiliations:** Department of Philosophy, Peking University, Beijing, China

**Keywords:** moral algorithm, experimental philosophy, is-ought problem, autonomous vehicle, Utilitarianism, Rawlsianism, Egoism, Hybrid approach

## Abstract

**Introduction:**

This study delves into the ethical dimensions surrounding autonomous vehicles (AVs), with a specific focus on decision-making algorithms. Termed the “Trolley problem,” an ethical quandary arises, necessitating the formulation of moral algorithms grounded in ethical principles. To address this issue, an online survey was conducted with 460 participants in China, comprising 237 females and 223 males, spanning ages 18 to 70.

**Methods:**

Adapted from Joshua Greene’s trolley dilemma survey, our study employed Yes/No options to probe participants’ choices and Likert scales to gauge moral acceptance. The primary objective was to assess participants’ inclinations toward four distinct algorithmic strategies—Utilitarianism, Rawlsianism, Egoism, and a Hybrid approach—in scenarios involving AVs

**Results:**

Our findings revealed a significant disparity between participants’ preferences in scenarios related to AV design and those focused on purchase decisions. Notably, over half of the respondents expressed reluctance to purchase AVs equipped with an “egoism” algorithm, which prioritizes the car owner’s safety. Intriguingly, the rejection rate for “egoism” was similar to that of “utilitarianism,” which may necessitate self-sacrifice.

**Discussion:**

The hybrid approach, integrating “Utilitarianism” and “Egoism,” garnered the highest endorsement. This highlights the importance of balancing self-sacrifice and harm minimization in AV moral algorithms. The study’s insights are crucial for ethically and practically advancing AV technology in the continually evolving realm of autonomous vehicles.

## 1 Introduction

In the past few years, we have witnessed autonomous vehicles (AVs) rise from concept to reality, offering an innovative solution to modern transportation issues. The advent of AVs brings with it the potential to drastically reduce human error, which is responsible for a significant number of road accidents (Sütfeld et al., 2017). Through precise sensors and AI algorithms, AVs may detect potential hazards and react to them faster than a human driver, leading to fewer collisions and ultimately saving lives. Moreover, in many cases, drivers prioritize self-preservation, often at the expense of pedestrians and other road users. AVs could offer a promising solution to mitigate this unjustified selfishness by replacing human decision-making with algorithms designed to prioritize overall safety and minimize harm. In addition, by utilizing optimized routing and platooning techniques, AVs are likely to alleviate traffic congestion in urban areas. Furthermore, AVs present an opportunity for increased mobility for groups such as the disabled or elderly, who may otherwise be unable to drive.

To further discuss AVs, it is essential to distinguish between the different levels of autonomy these vehicles can possess. The Society of Automotive Engineers (SAE) has defined a classification system which breaks down vehicle automation into six distinct levels, from 0 to 5.

Level 0 vehicles enjoy no automation, meaning all driving tasks are entirely performed by the human driver. Level 1 and 2 vehicles have some degree of driver assistance technology, like adaptive cruise control or automatic lane-keeping. From level 3 onward, vehicles assume a greater share of control. At level 3, or “conditional automation,” the vehicle can handle all aspects of driving in certain conditions, but a human must always be ready to take over. This requirement introduces a potentially risky transition period between automated and human driving that is not present in higher levels of automation. Level 4, or “high automation,” vehicles can operate without human input under specific conditions. The vehicle itself will handle all driving tasks and will not require human intervention in most scenarios. The highest level, Level 5, signifies full automation in all conditions. At this stage, the vehicle can handle all driving tasks under all environmental and traffic conditions. The vehicle is entirely self-driving and does not require human intervention at any point.

As we move toward higher levels of autonomy, these vehicles will inherently bear greater responsibility for making decisions on the road. At levels 4 and 5, AVs may encounter scenarios where they must make critical, split-second decisions on the road. Therefore, they have to face some complex moral dilemmas previously confined to philosophical discourse. The trolley problem, for instance, is a classic ethical dilemma that has now become a practical issue for AVs.

This conundrum involves a hypothetical situation where a trolley is on course to hit 5 people ahead, but a lever can divert the trolley to a different track where it would hit only one person (Foot, 1967; Bruers and Braeckman, 2014). The moral question here is whether it is better to actively cause one person’s death to save five. Similarly, an AV might have to decide between swerving to hit one person so as to avoid hitting 5 people ahead or continuing drive forward. These moral dilemmas highlight the need for the development of a “moral algorithm” to guide the decision-making process of AVs (Lucifora et al., 2020; Etienne, 2022; Himmelreich, 2022).

Given the absence of a definitive moral answer in dilemmas like the trolley problem, the quest for a universally applicable moral algorithm encounters similar challenges. In response to this challenge, many scholars delve into the realm of people’s moral preference in pursuit of the most widely accepted moral algorithm. Among these approaches, utilitarianism, advocating for the preservation of more lives at the expense of fewer, has garnered the highest support in previous surveys of trolley dilemma (Hauser et al., 2007; Rehman and Dzionek Kozłowska, 2018; de Melo et al., 2021). In dilemma scenario of AVs, scholars also observed that most participants showed a moral inclination toward utilitarianism, even when it entailed self-sacrifice (Bonnefon et al., 2016; Gawronski et al., 2017; Bergmann et al., 2018; Faulhaber et al., 2019). However, there are differences between people’s theoretical agreement with certain moral principles and their readiness to adopt these principles when faced with real-world consequences. Specifically, participants may agree, in principle, that it would be morally acceptable to sacrifice one passenger (including themselves) to save five passengers, a majority of participants indicated reluctance to purchase an AV programmed with a utilitarian moral algorithm (Bonnefon et al., 2016; Mayer et al., 2021).

These results revealed challenges to utilitarianism within the realm of AVs, prompting some scholars to propose the concept of “selfish” AVs that protect passengers, often referred to as Egoistic AVs (Coca-Vila, 2018; Liu and Liu, 2021). It is controversial whether egoism is a descriptive or normative concept, since Egoism can be viewed from both descriptive and normative angles. Descriptive egoism is often associated with psychological egoism. Psychological egoism is the idea that individuals, by nature, are inherently self-interested and that all of their actions are ultimately motivated by self-interest; while normative egoism, on the other hand, is a prescriptive ethical theory. It asserts that individuals ought to act in their self-interest, making self-interest a moral obligation or principle (Millán-Blanquel et al., 2020). However, the ambiguity surrounding this concept does not preclude its consideration as a viable algorithmic approach. The idea of an Egoistic algorithm gained traction as it directly addresses the preference for passenger protection. Mercedes-Benz, for instance, has remarked that their AVs would prioritize safeguarding the car’s passengers in collision situations (Leben, 2017, pp. 98–99). However, this approach met with significant criticism about prioritizing the lives of passengers over other vulnerable road users, such as children (Leben, 2017, p. 99). Moreover, there is also not enough empirical evidence that people would be willing to buy Egoistic AVs.

In addition to utilitarianism and egoism, the Rawlsian algorithm, proposed by Leben (2017), represents another noteworthy approach. This algorithm mainly relies on Maximin procedure, a strategy of maximizing the minimum payoffs. The core idea of the algorithm is to collect estimations of each player’s probability of survival for various potential actions, aiming to identify strategies that maximize overall survival rates. Nevertheless, this approach also received criticism about inequitable distribution of risks (Leben, 2017; Keeling, 2018). Additionally, the implementation of survival rate calculations cannot entirely eliminate the possibility of passenger self-sacrifice, which may, in turn, lead individuals to hesitate in selecting such an algorithm.

It is clear that Utilitarianism, Egoism, and Rawlsianism all faced their share of theoretical criticism, yet there has been no survey to directly compare their level of moral acceptance as well as preference of purchase, which is important in the previous research for discussing efficiency of moral algorithm. And it is worth noting that the moral acceptance of these algorithms, evidenced by theoretical agreement, may not seamlessly translate into consumer behavior (Bonnefon et al., 2016; Sui and Guo, 2020).

From the perspective of ethics, this distinction suggests that a moral algorithm based solely on “ought” principles prove inadequate, since people’s tendency of purchasing AVs may closely resemble an “is” problem, reflecting the actual preferences and behaviors of individuals, rather than a pure “ought” issue.^1^

Furthermore, this underscores the issue of personal perspective. Extensive research has established that individuals’ moral choices are notably influenced by their personal roles within a given moral dilemma (Hauser et al., 2007; Greene, 2016). Several investigations have delved into the specific perspectives of passengers, pedestrians, and observers in trolley dilemma, indicating people’s inclination to avoid moral algorithm involving self-sacrifice (Frank et al., 2019; Mayer et al., 2021). Nevertheless, it is essential to acknowledge that these perspectives exhibit disparities when compared to the traditional personal perspective encountered in the classic trolley problem.

In the classic version of the trolley problem where individuals are asked to make a decision about whether to pull a lever to divert a trolley and potentially save lives, people typically take on the role of an active decision-maker. From a personal perspective, individuals may see themselves as actively responsible for the consequences of their decision. They are not mere observers but are actively engaged in making a choice that directly impacts the outcome of the situation. And they are not directly involved as potential passengers or pedestrians who may be harmed by the consequences of their decisions. In the context of AVs, their personal perspective involves a sense of agency and moral responsibility for the potential harm or benefit that result from their decision. In other words, their role closely resembles that of the designers of moral algorithms, rather than passengers or pedestrians.

Therefore, this article aims to critically examine people’s preference of design and purchase among Utilitarianism, Egoism and Rawlsianism. Furthermore, we would also examine people’s reactions to a Hybrid algorithm that attempted to strike a balance between avoiding self-sacrifice and minimizing damage. Specifically, the Hybrid algorithm employs a dual approach, incorporating both a “driver-oriented” perspective and a “sacrifice the few to save the many” approach, which involves prioritizing the safety of passengers (car owners) in scenarios involving the passenger and pedestrians. However, when faced with pedestrian scenarios, the algorithm opts to safeguard the greater number of pedestrians by maneuvering the vehicle accordingly.

We expected that it would enhance people’s moral acceptance as well as inclination of purchase. Consequently, we aspire to make a meaningful contribution to the ongoing discourse surrounding the evolution of moral algorithms for AVs, thereby informing future endeavors within this swiftly advancing field.

## 2 Materials and methods

### 2.1 Participants

Experimental data was collected through Credamo, a reliable online third-party professional survey platform used to recruit participants. With the consent of anonymous participants, Credamo verified their location through their IP addresses to ensure that the data covered a majority of provinces in China. Participants in this study were presented with a brief online informed consent form that explained the anonymous nature of the research. They were informed that the study pertained to the ethical decision-making design of AVs and were encouraged to respond truthfully. They were also assured that there were no inherently right or wrong, good or bad answers, as their responses would be exclusively used for scientific research purposes. Furthermore, participants were explicitly informed that they had the option to exit the survey at any point, and the system would not retain or save their response records in such cases.

The final dataset consisted of 460 participants, including 237 females and 223 males. Participants’ ages ranged from 18 to 70. The age distribution within the sample is as follows: 44.13% of participants were between the ages of 18 and 30, 34.35% were between 31 and 40, and 11.52% were between 50 and 70. The original survey involved 480 participants. Each participant was compensated with 2 RMB, approximately equivalent to $0.3 USD, for their participation in the study. In order to maintain the reliability of the survey results, 20 participants were excluded from the data set, because 9 of them failed to provide a correct answer to the control question,^2^ while 11 of them completed survey less than 1 min, which is too fast to thoroughly read the vignettes.

### 2.2 Design

The survey was based on the trolley dilemma, a thought experiment often used in moral philosophy to explore ethical decision-making. This approach was chosen due to its use in previous studies on moral algorithms for AVs (Bonnefon et al., 2016; Leben, 2017; Bergmann et al., 2018). The experimental design was adapted from Greene’s classic survey on trolley dilemma, which employed Yes/No options to exam people’s choices and Likert scales to measure moral acceptance Greene (2016).

The survey aimed to assess participants’ tendency (design, purchase and moral acceptability) toward four distinctive moral algorithms (Utilitarianism, Rawlsianism, Egoism, and Hybrid approach) in scenarios of passenger-involved dilemma.

### 2.3 Procedure

Throughout the course of the experiment, we sequentially presented four different moral algorithms—Utilitarianism, Rawlsianism, Egoism, and Hybrid approach—replicating the order of occurrence in previous discussions and studies. To ensure participants’ focus and prevent distractions from other scenarios, we presented each scenario individually, allowing participants to engage with one scenario at a time. Participants were able to proceed to the next scenario after completing the previous one. The vignette presented a hypothetical moral dilemma:

*In the scenario of an autonomous driving accident, an autonomous car carrying one passenger (car owner) is speeding down the highway when suddenly 5 pedestrians appear ahead. The car’s brakes happen to fail at this moment. The way to save the 5 pedestrians is to swerve the car, but doing so will cause it to hit the guardrail, leading to either the death of the passenger or one pedestrian.*

Participants were asked to choose whether they would design (“Yes” or “No”) an AV according to the corresponding moral algorithm after reading the vignettes. They also need to rate their moral acceptability of the “yes” option for that algorithm on a 7-point scale ranging from 1 (“totally un-acceptable”) to 7 (“totally acceptable”). Moreover, they were asked to indicate whether they would like to purchase the AV with this algorithm (“Yes” or “No”). At the end of the survey, they were ask to choose which one they would like to purchase among Utilitarianism, Rawlsianism, Egoism, and the Hybrid AVs.^3^

In Utilitarian scenario, participants would be asked that as designers whether they would adopt a “sacrifice the few to save the many” strategy in the vehicle design, allowing the autonomous car to sacrifice a passenger (car owner) or a pedestrian in this scenario.

In Rawlsian scenario, participants, as designers, need to decide whether to adopt a “minimize maximum fatality rate” strategy, that is, if the “death rate of 5 people is 90% when driving forward, and the car owner’s death rate is 10%; while the death rate of 5 people is 10% when swerve, and the car owner’s (or pedestrian’s) death rate is 80%,” then choose to swerve because it results in a lower maximum death rate. Conversely, if the “death rate of 5 people is 80% when driving forward, and the car owner’s (or pedestrian’s) death rate is 10%; while the death rate of 5 people is 10% when swerve, and the car owner’s death rate is 90%,” then choose to drive forward.^4^ In Egoistic scenario, participants need to consider whether design a “driver-oriented” strategy, which directly sacrificing pedestrians to protect the passenger (car owner) in the event of an accident.

In Hybrid scenario, participants would choose whether to design a combined strategy of “driver- oriented” and “sacrifice the few to save the many.” This combined strategy means that in scenarios involving the passenger (car owner) and pedestrians, priority is given to protecting the passenger (car owner), while in pedestrian scenarios, a choice is made to protect the majority of pedestrians by swerve the vehicle.

In addition to evaluating the moral acceptability and participants’ tendency to purchase an AV implementing various moral principles, participants were also asked to choose among Utilitarianism, Rawlsianism, Egoism, and the Hybrid algorithm when deciding which one they would purchase.

## 3 Results

To address our previous questions and analyze the collected data, we used SPSS 20 for all analyses and implemented an array of statistical methods, including the Chi-square test, *t*-test, and ANOVAs on our collected data. Additionally, we applied Bonferroni correction and performed the Kruskal-Wallis test with *post-hoc* comparisons.^5^

The most prominent finding emerging from this statistical analysis was the preference of participants for the Hybrid algorithm. The data showed that this algorithm was not only preferred, but also perceived as the most morally acceptable choice, indicating a convergence of pragmatic decision-making and ethical comfort. Beyond general moral acceptability, another interesting observation was noted in the behavior of those who choose to design or purchase specific AVs. These participants demonstrated a high degree of moral acceptability compared with those who opted against purchasing.

### 3.1 Discrepancy of design and purchase

**Figure 1:**
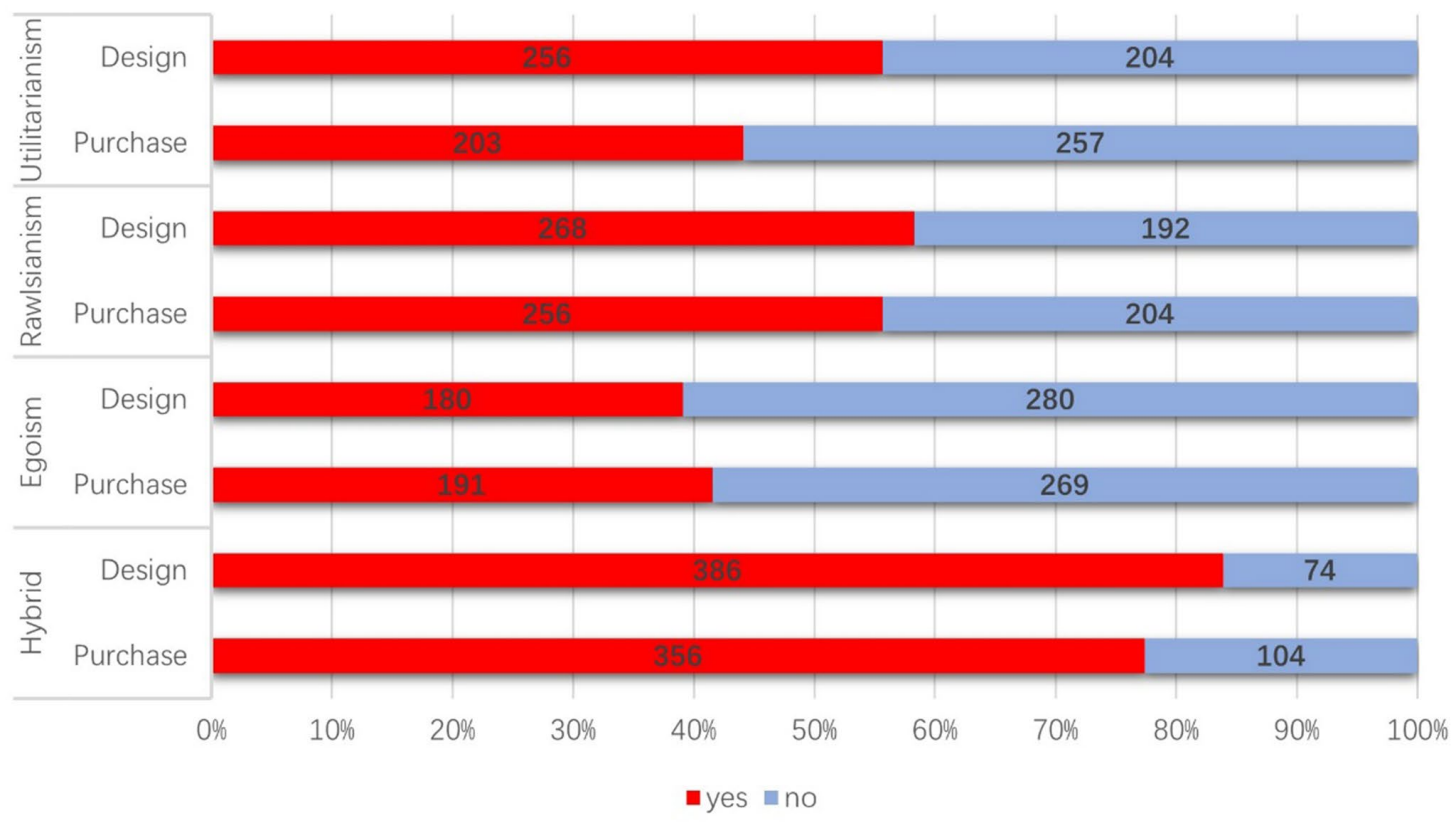
Choices of design and purchase.

In Hybrid scenario, a majority of participants (83.91%) expressed their preference for designing an AV using this approach. Furthermore, a significant proportion (77.39%) indicated their intention to purchase an AV designed in accordance with this approach. However, in Rawlsian scenario, the percentage of participants who chose to design an AV using this approach decreased to 58.26%. Similarly, the proportion of participants who expressed their willingness to purchase an AV based on the Rawlsian design dropped to 55.65%. Utilitarianism approach exhibited even lower ratios, with only 55.65% of participants indicating their preference for designing an AV based on this ethical framework, and a mere 44.13% expressing their intention to purchase an AV designed according to Utilitarian principles. Notably, Egoism approach had the lowest percentages among all scenarios. Only 39.13% of participants favored designing an AV based on Egoism, and 41.52% expressed their likelihood of purchasing an AV designed with an Egoistic perspective in mind (see Figure 1).

In the design scenario, a Chi-square test of independence was conducted to examine the differences among various moral algorithms and applied the Bonferroni correction for multiple comparisons (α = 0.05). We found that the association among four algorithms is significant, with the effect size indicating a small but statistically significant effect, *χ*^2^ (3, *N* = 1840) = 195.65, *p* < 0.0001, φ = 0.33. Subsequently, we conducted pairwise comparisons between the groups to identify specific differences, revealing significant variations with the exception of the comparison between “Rawlsianism and Egoism” (see Table 1).

**Table 1:** Chi-Square results of design and purchase.

	Design			Purchase		
	*χ*^2^ (1,460)	*p*	φ	*χ*^2^ (1,460)	*p*	φ
Hybrid vs. Utilitarianism	21.57	<0.0001	0.22	28.77	<0.0001	0.25
Hybrid vs. Rawlsianism	13.19	<0.0001	0.17	39.13	<0.0001	0.29
Hybrid vs. Egoism	19.44	<0.0001	0.21	46.61	<0.0001	0.32
Utilitarianism vs. Rawlsianism	57.53	<0.0001	0.35	170.22	<0.0001	0.61
Utilitarianism vs. Egoism	18.18	<0.0001	0.2	0.39	0.57	0.03
Rawlsianism vs. Egoism	0.37	0.56	0.03	4.97	0.029	0.1

Similarly, in the purchase scenario, significant differences of Chi-square results were observed among the moral algorithms, *χ*^2^ (3, *N* = 1840) = 148.72, *p* < 0.0001, φ = 0.28, Bonferroni-corrected. Through pairwise comparisons among the groups, we identified significant differences in all cases except for the “Utilitarianism and Egoism” comparison. Gender showed no significant differences in both design and purchase scenarios.

These results emphasize the influence of moral algorithms on individuals’ decision-making processes in both the design and purchase of AVs, highlighting the need for further investigation into the underlying factors driving these preferences and the implications for ethical decision-making in autonomous systems (see Table 1).

An important Chi-square result worth mentioning is the observed asymmetrical relationship between individuals’ tendencies to design or purchase AV within certain scenarios. The data revealed distinct patterns in participants’ preferences, indicating a notable difference in their decision-making processes. Participants exhibited a greater inclination toward design in the contexts of Utilitarianism, Rawlsianism, and Hybrid, while demonstrating a higher preference for purchasing in scenario of Egoism (see Table 2).

**Table 2:** Chi-Square results of design vs. purchase.

	*χ*^2^ (1,460)	*p*	φ
Utilitarianism design vs. purchase	165.32	<0.0001	0.6
Rawlsianism design vs. purchase	138.57	<0.0001	0.55
Egoism design vs. purchase	248.21	<0.0001	0.74
Hybrid design vs. purchase	223.42	<0.0001	0.7

Upon evaluating participants’ preferences among four proposed moral algorithms, the Hybrid algorithm proved to be the most popular choice, with a striking 68.91% of participants expressing a preference to purchase vehicles utilizing this moral approach. A deep dive into the category “I have other answers” selected by 45 participants reveals a diverse set of perspectives and reservations regarding AVs adoption.

**Figure 2:**
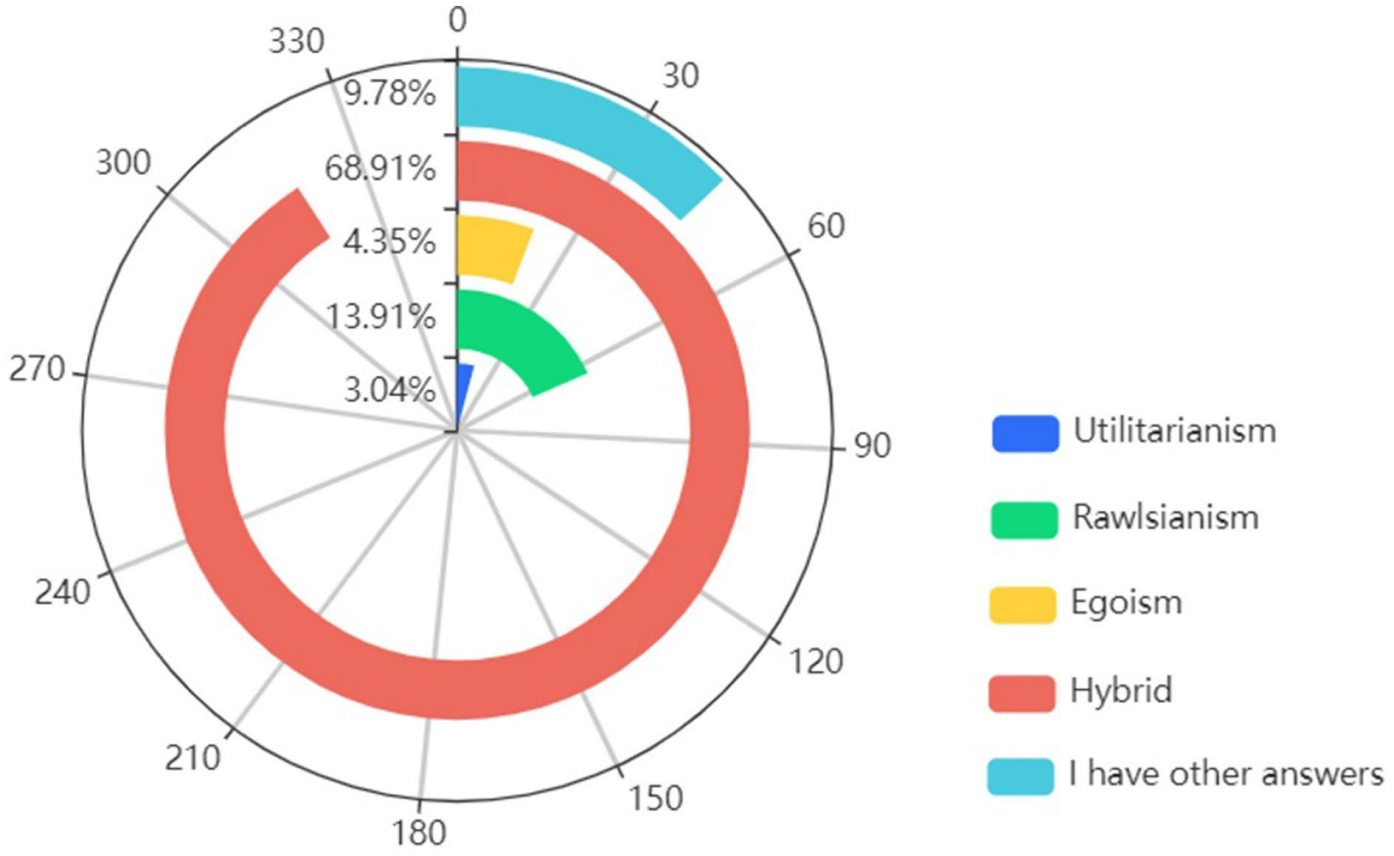
People’s preferences of purchase among all moral algorithm.

Among these participants, 18 showed a reluctance to purchase AVs, highlighting a degree of apprehension about this nascent technology. Meanwhile, 11 participants suggested that AVs should be designed to avoid these ethical dilemmas altogether, either by adopting a slower driving speed or by engineering improvements to reduce the failure rate of the vehicles. An equal number, 11 participants, expressed a desire for alternative solutions that do not involve any sacrifice, emphasizing the need for innovative strategies to navigate complex ethical scenarios. The rest 5 participants argued for non-interference in dilemma scenarios, implying a preference for a hands-off approach (see Figure 2).

### 3.2 Differences of moral acceptability

In terms of moral acceptability, the Hybrid received the highest rating on a scale ranging from 1 to 7, with a mean score of 5 and a standard deviation (SD) of 1.62. This indicates that participants perceived the Hybrid approach to be the most morally acceptable among the presented algorithms.

Following the Hybrid, the Rawlsianism received a slightly lower mean score of 4.28, with a SD of 1.7. Participants seems to consider it to be relatively morally acceptable, but to a lesser extent compared to the Hybrid.

Similarly, the Utilitarianism garnered a mean score of 3.97, with a SD of 1.85. Participants perceived this approach to be less morally acceptable compared to the previous two approaches.

**Figure 3:**
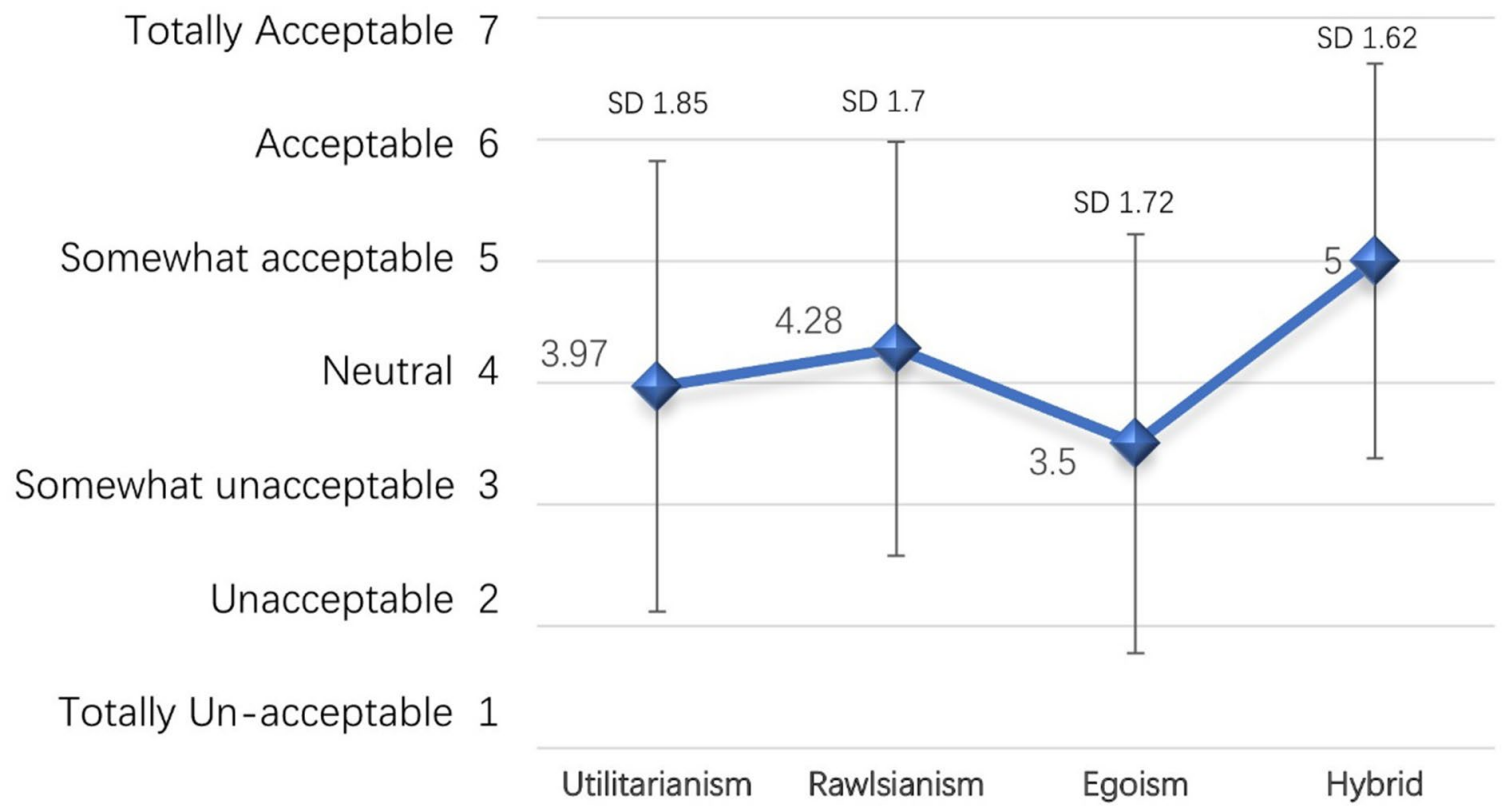
Moral acceptability among moral algorithm.

Among them, the Egoism received the lowest mean score of 3.5, with a SD of 1.72. This indicates that participants thought the Egoism approach to be the least morally acceptable among the presented scenarios (see Figure 3).

**Figure 4:**
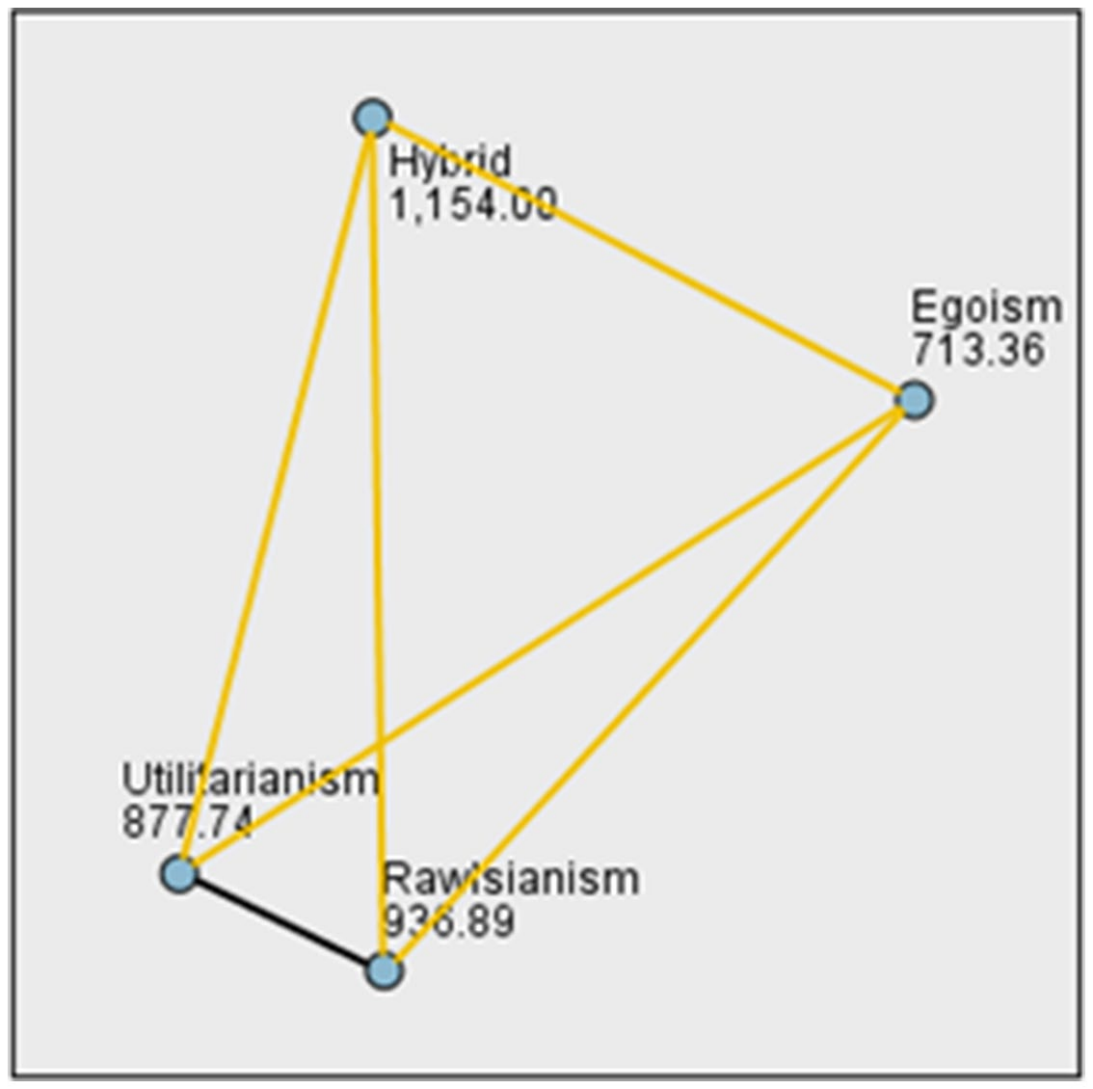
The sample average rank of Kruskal–Wallis test.

Gender was found to have no significant impact on difference in moral acceptance. In the analysis of differences among the four groups, we conducted a one-way ANOVA to assess the presence of differences among the four scenarios, which yielded a significant main effect of different moral algorithms, *F*(3, 1836) = 58.92, *p* < 0.0001, η^2^_p_ = 0.09, indicating a medium effect size^6^. Additionally, we performed a Kruskal-Wallis *post-hoc* test, which also yielded a statistically significant result, *H*(3, 1840) = 167.35, *p* < 0.0001, confirming the presence of differences among the scenarios. And significant differences were observed in all cases except for the “Utilitarianism and Rawlsianism”(see Table 3 and Figure 4). These findings indicate substantial variations in individuals’ moral preferences toward these different moral algorithms.

**Table 3:** Kruskal–Wallis test results of moral acceptability.

	*H* (3,1840)	*p*
Hybrid vs. Utilitarianism	276.26	<0.0001
Hybrid vs. Rawlsianism	217.11	<0.0001
Hybrid vs. Egoism	440.64	<0.0001
Utilitarianism vs. Rawlsianism	59.15	0.52
Utilitarianism vs. Egoism	164.38	<0.0001
Rawlsianism vs. Egoism	223.53	<0.0001

The observed significant differences suggest that individuals’ moral judgments and decision-making processes significantly diverge when evaluating and selecting among these moral algorithms. This implies that individuals hold distinct moral preferences and principles when it comes to assessing the ethical frameworks underlying these algorithms. The presence of large effect sizes further emphasizes the magnitude of these variations, highlighting the substantial impact of the choice of moral algorithm on individuals’ moral judgments and decision-making.

**Figure 5:**
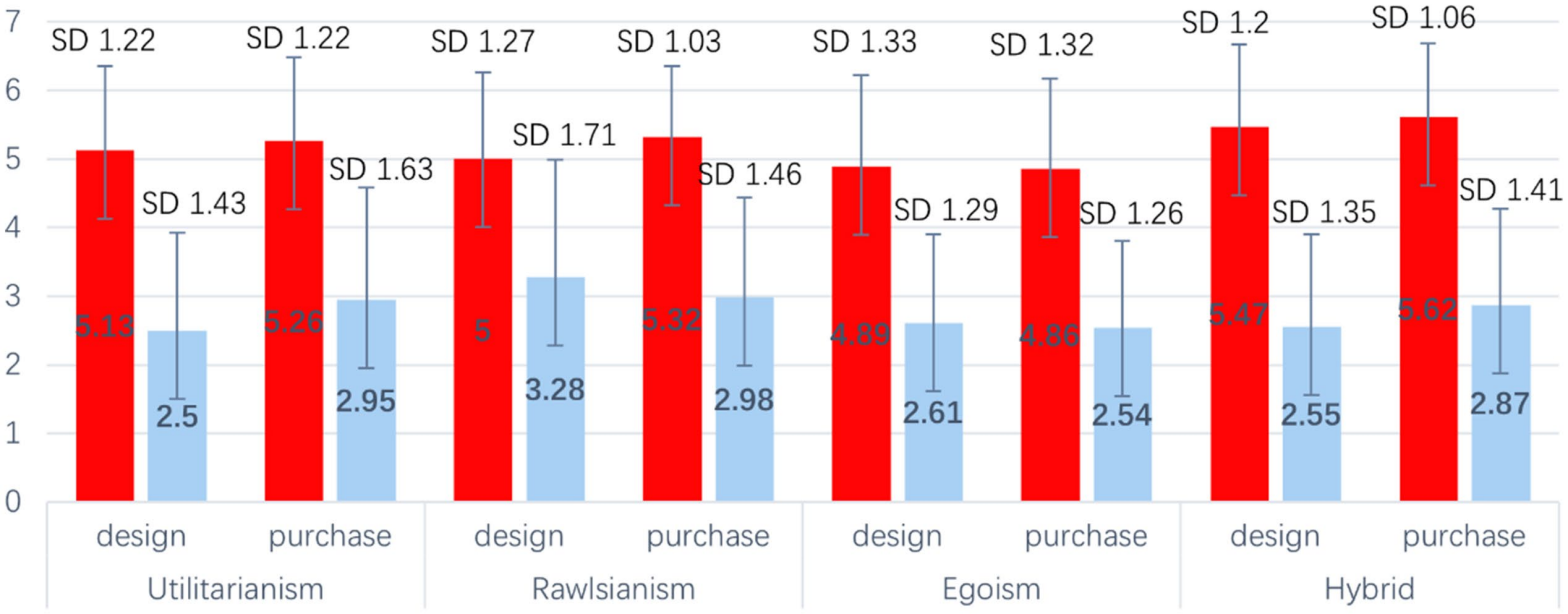
Moral acceptability of different choices.

Our analysis also examined the relationship between individuals’ moral acceptability ratings and their choices regarding the design and purchase of specific moral algorithms within different scenarios (see Figure 5).

We performed a series of one-way analysis of variance (ANOVA) tests to assess the influence of two independent factors: “design” and “purchase,” on the levels of moral acceptance associated with four distinct moral algorithms. These ANOVA analyses were conducted separately for each independent factor. We found a consistent pattern where individuals who chose to design a moral algorithm within a particular scenario demonstrated higher moral acceptability toward that specific moral algorithm. The same pattern emerged in relation to the scenario of purchase.

According to the results of one-way ANOVAs, individuals who chose to design or purchase specific AVs exhibited significantly higher levels of moral acceptability compared to those who did not engage in these activities. Furthermore, the effect size, as measured by partial eta squared (η^2^_p_), was found to be substantial. This suggests that the influence of choosing to design or purchase certain AVs on moral acceptability is not merely negligible, but rather carries considerable importance (see Table 4).

**Table 4:** One-way ANOVA of moral acceptability.

	Design vs. not design			Purchase vs. not purchase		
	*F* (1, 459)	*p*	η^2^_p_	*F* (1, 459)	*p*	η^2^_p_
Utilitarianism	102.1	< 0.0001	0.18	282.32	< 0.0001	0.38
Rawlsianism	154.57	< 0.0001	0.25	405.75	< 0.0001	0.47
Egoism	334.86	< 0.0001	0.42	363.91	< 0.0001	0.44
Hybrid	351.44	< 0.0001	0.43	464.97	< 0.0001	0.50

## 4 Discussion

In our study, we found significant disparities among the four moral algorithms (Utilitarianism, Rawlsianism, Egoism and Hybrid). Specifically, people did not tend to favor Utilitarianism or Rawlsianism, which would sacrifice the passenger to save five pedestrians, nor did they prefer Egoism that would sacrifice five pedestrians for the sake of the passenger. Additionally, we observed a strong preference for the Hybrid algorithm among our participants. This finding suggested that the Hybrid algorithm, which strives to strike a balance between avoiding self-sacrifice and minimizing overall harm, resonated strongly with individuals in our study. Furthermore, we noted a notable distinction between the design and the purchase scenario, indicating differences in people’s tendencies based on their personal perspectives.

These findings highlight the complexities of human moral reasoning and the difficulties of designing moral algorithms for AVs. In the following discussion, we will delve into the implications of these findings, to clarify the role of morality in moral algorithms.

### 4.1 The rejection of egoism

In the survey we conducted, the moral factor still plays an important role in people’s preference of moral algorithm. The most striking evidence is our respondents’ clear disinclination toward an Egoistic algorithm. Based on previous studies, there exists a hypothesis that consumers might find egoism more acceptable in comparison to moral algorithms that entail self-sacrifice (Leben, 2017). However, this hypothesis is over-simplified. In other words, if companies such as Mercedes-Benz design Egoistic AVs, they would not only face moral criticism but also practical rejection.

There may be several reasons for this. Firstly, the principles of egoism, while beneficial to individuals, can lead to outcomes that are widely perceived as unfair or morally wrong, since it potentially results in harm or even the death of pedestrians in certain scenario. This may conflict with deeply held values of fairness and the equal value of all lives. Although some philosophers, such as Ayn Rand, argued that egoism is a morally defensible position (Badhwar, 1993), it still showed the lowest rate of moral acceptability in our survey, in other words, egoism is widely considered as morally wrong when appealing to folk intuition^7^. Furthermore, the purchase rate of Egoistic vehicles does not exhibit significant differences when compared to Utilitarian AVs, potentially implying a willingness to accept self-sacrifice.

Secondly, people may worry about the social implications of widespread adoption of Egoistic algorithms. If all AVs were programmed to protect their passengers at all costs, this could lead to a breakdown of trust and cooperation on the roads, resulting in more harm overall. The widespread adoption of Egoistic algorithms could engender a form of vehicular “social Darwinism”, where the strongest – in this case, those within the safety of their AVs – survive, while the most vulnerable road users are left to bear the brunt of any harm. This, in turn, could foster a climate of fear and mistrust, with pedestrians and other road users constantly wary of AVs programmed to prioritize their own safety over that of others. Moreover, a broad acceptance of Egoistic algorithms could potentially destabilize the cooperative equilibrium that currently exists. By effectively sanctioning a disregard for the safety of others, we are going to cultivate an environment where each vehicle is solely out for itself. Those would potentially lead to a more chaotic and dangerous situation.

Thirdly, the disapproval of the Egoistic algorithm may also reflect considerations for moral consistency. Generally, the notion of moral consistency involves maintaining the same moral principles and standards across different contexts and situations. In the context of the Egoistic algorithm for AVs, moral consistency implies that if one endorses the principle of self-preservation at all costs for themselves, they should also accept it when it is applied by others. However, this leads to a paradoxical situation. On the one hand, individuals demand to prioritize their own safety as passengers of AVs. On the other hand, they should understand AVs to prioritize passengers and to disregard their safety when they are pedestrians. This conflict may lead to cognitive dissonance, a psychological discomfort when one’s beliefs or attitudes contradict one another (Festinger, 1962). In this case, it is the contradiction between an individual’s self-preservation instincts and their understanding of fairness and reciprocity. To resolve this dissonance, people are likely to reject the Egoistic algorithm to maintain moral consistency.

Fourthly, the act of consciously choosing such a vehicle could be viewed as endorsing this moral stance, which essentially amounts to disregarding the wellbeing of others in favor of one’s own safety. This endorsement could somehow make the individual morally culpable for the harm caused to others. In other words, by choosing an Egoistic AV, individuals may feel they are implicitly agreeing to the potential harm that may be caused by vehicle. This increased moral responsibility could be a significant deterrent for many people. Most individuals are likely to be uncomfortable with the idea of being morally responsible for harm caused to others, especially when such harm is a direct consequence of prioritizing their safety.

To sum up, the Egoistic algorithm for AVs, while it may provide immediate self-protective benefits, proves to be deeply contentious. Although the appeal of self-preservation is undeniable, our findings suggest that individuals value broader ethical considerations. These considerations encompass fairness, societal harmony, moral consistency, and the avoidance of direct moral culpability, all of which highlight the complex moral landscape within which the AVs of the future must operate.

### 4.2 The preference of hybrid approach

The preference for a hybrid approach, as demonstrated by the majority of our survey respondents, indicates the ethical complexities involved in the moral algorithms of AVs. People, in general, strive to balance competing moral considerations rather than subscribe to any single principle in its entirety. The Hybrid algorithm leans toward egoism, prioritizing passenger safety in danger scenarios, and adopts a utilitarian approach when scenarios only involve pedestrians. Although both Egoism and the Hybrid method share an element of self-preservation, the Hybrid approach seems to avoid the perception of complete disregard for others’ lives.

By integrating Utilitarian principles when scenarios primarily involve pedestrians, Hybrid method tries to represent a balance between self-preservation and the welfare of others. This perceived fairness, even if it does not come into play in passenger-danger scenarios, could lead to more acceptance among people. The Egoistic algorithm, by always prioritizing passenger safety, may be seen as overly self-interested and potentially unfair, leading to less approval.

Moreover, the Hybrid algorithm is adaptive and takes into account the specifics of each scenario. While it does prioritize passengers when they are in danger, it also considers the greater good in other situations. This context-aware decision-making might be seen as more intelligent and morally sound, although the differences between the Hybrid and Egoism may be relatively small.

The perception of morality indeed could contribute to the preference for the Hybrid algorithm. When people imagine themselves as pedestrians, they might perceive the Hybrid algorithm as being more favorable or less threatening compared to the Egoistic algorithm. This is due to the fact that the Hybrid algorithm, incorporating principles of utilitarianism, would aim to minimize overall harm in scenarios involving only pedestrians, which the Egoism shows no concern about it. Therefore, as pedestrians, individuals might feel safer or more protected knowing that the AVs on the road would attempt to cause the least overall harm. The expectation of greater safety, whether statistically significant or not, could potentially make the Hybrid algorithm more acceptable.

In this sense, the preference for the Hybrid algorithm can be seen as a compromise between one’s personal safety and the broader ethical concern for the wellbeing of others. Moreover, the preference for the Hybrid algorithm might presented a paradox of moral psychology that Philippa Foot indicated. According to Foot, people can appreciate and recognize moral virtues such as courage, generosity, or self-sacrifice, acknowledging them as desirable traits. Yet, recognizing a virtue does not necessarily mean embodying it themselves, an example is that someone may admit that they are coward while still having no intention to become more courageous (1969, p.209). In the case of moral algorithm, some people are essentially acknowledging the virtue of self-sacrifice for the greater good, which is a key aspect of utilitarian philosophy. Yet, they also desire the assurance that their own safety will be prioritized when they are passengers in an AV. In a sense, they recognize the virtue of self-sacrifice but also appreciate the necessity of self-preservation.

From a broader perspective, the preference for the Hybrid algorithm reveals a sort of moral compromise. People seem to understand the ethical virtues of minimizing overall harm and self-sacrifice, but at the same time, they retain the instinct for self-preservation. In this way, the Hybrid algorithm encapsulates a reconciliation of these conflicting impulses, offering a solution that seems to be both practically appealing and morally defensible.

The popularity of the Hybrid algorithm suggests that, in the context of decision-making, people may be more comfortable with a pragmatic approach that balances ethical considerations with personal safety. This aligns with Foot’s observation that recognition of a virtue does not necessarily lead to the adoption of that virtue in one’s own life. In both cases, there is a tacit acknowledgment of the complexities and contradictions inherent in moral decision-making. The appeal of the Hybrid algorithm, underscores the importance of balancing self-sacrifice and minimizing harm considerations in the design and implementation of moral algorithms for AVs.

It is evident that people are attracted to an algorithm that manages to integrate both self-preservation instincts and a broad commitment to minimizing harm and promoting the greater good. This balance reflects a pragmatic approach to the design of moral algorithms. It suggests that algorithm developers should not simply aim to replicate human moral decision-making or instinctive reactions in crisis situations, nor should they exclusively follow a specific moral philosophy in a rigid, uncompromising manner. Instead, they should seek to blend these perspectives in a way that respects the complexity and diversity of human moral judgment. The acceptance of AVs by society will likely depend on whether people perceive their decision-making algorithms as reflecting both the practical realities of self-preservation and the broader ethical principles that they value.

### 4.3 The differences between design and purchase

One of the key findings of our study pertains to the significant differences observed between individuals’ perceptions of design choices and their intentions to purchase AVs programmed with specific moral algorithms.

From the perspective of moral psychology, it may be explained by Joshua Greene’s dual-process theory of moral judgment. Greene’s theory suggested that our moral decisions stem from one of two distinct cognitive processes, namely, automatic-emotional process and conscious-controlled process (Greene, 2009). The automatic-emotional process is rapid and unconscious, leading to intuitive actions and judgments, while the conscious-controlled process is slower and involves deliberate reasoning. When people are confronted with a low-conflict impersonal dilemma (such as Trolley problem), they are more likely to make a utilitarian judgment (sacrifice the few to save the many), which supported by conscious-controlled process. However, in a high-conflict personal dilemma (such as Footbridge problem^8^), most people would refuse to sacrifice one person to save five (Greene, 2014). Briefly, Greene’s theory shows that people’s deed and deliberative moral judgments can be sometimes conflict with each other.

In the purchase scenarios of AVs that involves self-sacrifice, we can observe that the situation shares similarities with high-conflict personal moral dilemmas, akin to the well-known footbridge problem. In these scenarios, individuals are more likely to confront the prospect of self-sacrifice to save others, a situation that tends to evoke powerful emotional responses deeply tied to self-preservation. This heightened emotional involvement often leads individuals to resist taking the utilitarian action, where the sacrifice of one for the benefit of many is deemed necessary.

However, in the design scenarios, participants are in a distinct cognitive position compared to the purchase scenarios. When they engage in the design of moral algorithms for AVs, they adopt a role that aligns more closely with the conscious-controlled process in Joshua Greene’s dual-process theory of moral judgment (Greene, 2009). In this deliberative role, individuals may tend to consider the broader ethical implications and principles behind their decisions. Unlike the emotional responses that may be triggered when envisioning themselves as potential passengers or pedestrians facing self-sacrifice, the design role prompts participants to reflect on abstract ethical principles and trade-offs. They are not directly confronted with the immediate emotional turmoil associated with self-preservation, as they are not the ones who would personally experience the consequences of their algorithmic choices.

As a result, the design scenario allows individuals to make decisions that may prioritize utilitarian principles more readily. In this context, they can think about optimizing overall outcomes and minimizing harm from a detached, ethical standpoint without the emotional burden of envisioning personal sacrifice. This cognitive shift between the purchase and design scenarios underlines the complexity of moral decision-making in the realm of AVs.

This divergence highlights a crucial aspect of ethical decision-making in the context of AVs: individuals contemplating the design of moral algorithms may have significant different preferences compared to consumers. It is worth emphasizing that the purpose of designing AVs extends beyond creating a morally acceptable AV; it encompasses designing a vehicle that people are willing to purchase and use in their daily lives.

This emphasis on consumer acceptance and utilization is crucial, as the effectiveness of AVs in enhancing road safety depends on their adoption and active use by individuals. Therefore, it is not sufficient for them to be ethically sound on paper; they need to gain the trust and approval of potential users. It is when these vehicles are readily embraced by consumers and integrated into their daily routines that they can truly contribute to a safer and more secure traffic environment.

To sum up, the focus on consumer acceptance and utilization is integral to the ethical considerations surrounding AVs. It emphasizes the need for a holistic approach that combines moral acceptability with practical desirability. Only when these two facets align can AVs truly fulfill their potential in making our roads safer and reducing accidents.

## 5 Limitation and future research

While this study has yielded valuable insights into the ethical implications of AVs, it is important to recognize and address several limitations.

Firstly, our research primarily relied on presenting participants with vignettes illustrating moral dilemmas. While this approach provides a controlled and systematic exploration of ethical decision-making, it may not fully capture the complexity of real-world scenarios. Future investigations could enhance the ecological validity of research by employing immersive technologies like Virtual Reality (VR). Utilizing VR could offer participants a more lifelike and emotionally engaging experience, potentially yielding results that better mirror real-world decision-making processes.

Secondly, our study aimed to provide a comprehensive comparison of four distinct moral algorithms, with equal effort dedicated to describing the four scenarios. Nevertheless, we acknowledge that the Rawlsian maximin procedure employed in our study can be quite intricate, potentially leading to challenges in participants’ full comprehension. Specifically, some individuals may not have completely grasped that this algorithm does not consider the number of individuals at risk but instead prioritizes minimizing the worst-case outcome based on death probability rates. Recognizing this limitation, we are committed to addressing it in our future research endeavors. We intend to incorporate these valuable insights into our upcoming studies. In particular, we plan to conduct a follow-up study that delves deeper into the comprehension and interpretation of the Rawlsian maximin procedure, using scenarios that involve just one driver and one pedestrian.

Thirdly, it is essential to acknowledge that this article specifically concentrated on moral dilemmas within the context of AVs. Although these dilemmas are undoubtedly a crucial facet of the ethical landscape surrounding AVs, they represent only a subset of the broader challenges and considerations associated with this emerging technology. AVs introduce a multitude of legal, regulatory, safety, and societal issues that extend beyond the realm of moral dilemmas. Future research endeavors should continue to explore these multifaceted aspects, contributing to a comprehensive understanding of the ethical dimensions of AVs.

## 6 Conclusion

Our study reveals that individuals demonstrate a preference for a Hybrid algorithm, which attempts to harmoniously balance these elements. This reflects a blend of the practical need for self-preservation with the moral imperative to minimize harm and promote the common good. The intricate equilibrium seems to resonate with the general public, providing a morally robust framework for decision-making in AVs.

Taking into consideration the various levels of autonomy in vehicles, from no automation at Level 0 to full automation at Level 5, the need for this balance in moral algorithms becomes even more pronounced. As the level of autonomy increases, and assume greater responsibility for decision-making, the integration of moral and ethical considerations within their operating algorithms becomes crucial. Neglecting this crucial balance could result in systems that are either overly utilitarian or excessively self-interested, both of which could alienate users and result in societal backlash. A skewed prioritization toward either extreme could undermine the acceptance and adoption of AVs, hampering the potential benefits they offer in terms of safety, efficiency, and sustainability.

Therefore, an understanding of this balance is not simply a philosophical or academic thought experiment, but a practical necessity for the successful integration of AVs into society. Recognizing and addressing this challenge would be a key step toward the safe and ethical use of AVs in our everyday lives. Therefore, algorithm designers and policymakers must work together to ensure that the choices made by AVs mirror both the reality of human instincts and our moral expectations. By maintaining a balanced approach in moral algorithms, AVs can navigate complex moral dilemmas while promoting safety, fairness, and public trust. Such an approach ensures that the societal benefits of autonomous technologies are realized while upholding fundamental ethical principles and accommodating diverse perspectives.

## Data availability statement

The datasets presented in this study can be found in online repositories. The names of the repository/repositories and accession number(s) can be found below: https://osf.io/nkvjc/?view_only=7ec86c4c97154e22bd7083a2e5e52e25.

## Ethics statement

The studies involving humans were approved by the Association for Ethics and Human and Animal Protection of the School of Psychological and Cognitive Sciences of Peking University. The studies were conducted in accordance with the local legislation and institutional requirements. The participants provided their written informed consent to participate in this study.

## Author contributions

The author confirms being the sole contributor of this work and has approved it for publication.

## Footnotes

^1^By using the word “ought” and “is,” we are using David Hume’s “is-ought” concept, which suggests that there are fundamental differences between prescriptive statements (what “ought” to be) and descriptive statements (what “is”). Normally, a moral judgment involves prescriptive statements, prescribing what ought to be done in a given situation rather than what actually would be done.

^2^In this survey, the control question is to ask people choose number 1 from the following numbers.

^3^In order to prevent potential biases or preconceptions associated with these terms from influencing participants’ responses. We consciously avoided using terms such as “utilitarianism,” “egoism,” “Rawlsianism,” or “Hybrid” to describe the moral algorithms that presented to participants in our survey. However, for the sake of brevity and clarity, we would use these terms to summarize the feature of each scenario in this paper.

^4^For more details of Rawlsian algorithm see (Leben, 2017).

^5^The vignettes of the survey are given in an Appendix in Supplementary material to this article. Appendix and research data can be found in https://osf.io/nkvjc/?view_only=7ec86c4c97154e22bd7083a2e5e52e25

^6^For partial eta squared, Cohen suggested that 0.01 be considered a “small” effect size, 0.06 represents a “medium” effect size and 0.14 a “large” effect size.

^7^The term “appeal to folk intuition” is an approach of experimental philosophy, which refers to using common or widespread intuitive beliefs or judgments that people tend to have about specific philosophical concepts. This approach is a departure from traditional philosophy, which often involves the use of thought experiments or reasoned arguments largely disconnected from empirical data. In contrast, experimental philosophers gather data through surveys and experiments to understand what intuitions people actually have, rather than relying on their own introspective analysis.

^8^Footbridge problem is a thought experimental that can be considered as an updated version of Trolley problem. In this scenario, a is hurtling down a track toward five people. The only way to stop it is by pushing a very heavy man off a bridge under which the trolley will pass. This action would inevitably kill the man but save the five people on the track. According to previous researches, more people would choose a utilitarian method in trolley case, while most would refuse to do so in footbridge case (Hauser et al., 2007).
