# Supplementary material for: Exploring moral algorithm preferences in autonomous vehicle dilemmas: an empirical study
Source: Front Psychol. 2023 Nov 28;14:1229245. doi: 10.3389/fpsyg.2023.1229245 (PMC10713701; doi:10.3389/fpsyg.2023.1229245)
Supplement: Supplementary file 1 [file Data_Sheet_1.docx]

### Vignettes for survey

*Scenario of Utilitarianism*

In the scenario of an autonomous driving accident, an autonomous car carrying one passenger (car owner) is speeding down the highway when suddenly 5 pedestrians appear ahead. The car's brakes happen to fail at this moment. The way to save the 5 pedestrians is to swerve the car, but doing so will cause it to hit the guardrail, leading to either the death of the passenger or one pedestrian.

In the scenario of the autonomous driving accident, if the car continues drive forward, it may kill five pedestrians, while turning might kill one pedestrian or possibly the car owner.

If you were a designer, would you adopt a "sacrifice the few to save the many" strategy in the vehicle design, allowing the autonomous car to sacrifice a passenger (car owner) or a pedestrian in this scenario?

Yes No

In this scenario, what is your level of moral acceptance of the "sacrifice the few to save the many" approach?

(1) Totally acceptable

(2) Acceptable

(3) Somewhat acceptable

(4) Neutral

(5) Somewhat unacceptable

(6) Unacceptable

(7) Totally unacceptable

If such a "sacrifice the few to save the many" autonomous car was introduced to the market, would you consider buying it?

Yes No

*Scenario of Rawlsianism*

In the scenario of an autonomous driving accident, an autonomous car carrying one passenger (car owner) is speeding down the highway when suddenly 5 pedestrians appear ahead. The car's brakes happen to fail at this moment. The way to save the 5 pedestrians is to swerve the car, but doing so will cause it to hit the guardrail, leading to either the death of the passenger or one pedestrian.

In the scenario of the autonomous driving accident, if the car continues drive forward, it may kill five pedestrians, while turning might kill one pedestrian or possibly the car owner.

If you were a designer, would you consider adopting a "minimize maximum fatality rate" strategy, that is, if the "death rate of 5 people is 90% when driving forward, and the car owner's death rate is 10%; while the death rate of 5 people is 10% when swerve, and the car owner's (or pedestrian's) death rate is 80%", you would choose to swerve because it results in a lower maximum death rate. Conversely, if the "death rate of 5 people is 80% when driving forward, and the car owner's (or pedestrian's) death rate is 10%; while the death rate of 5 people is 10% when swerve, and the car owner's death rate is 90%", you would choose to drive forward.

Yes No

In this scenario, what is your level of moral acceptance of the "minimize maximum fatality rate" approach?

(1) Totally acceptable

(2) Acceptable

(3) Somewhat acceptable

(4) Neutral

(5) Somewhat unacceptable

(6) Unacceptable

(7) Totally unacceptable

If such a " minimize maximum fatality rate" autonomous car was introduced to the market, would you consider buying it?

Yes No

*Scenario of Egoism*

In the scenario of an autonomous driving accident, an autonomous car carrying one passenger (car owner) is speeding down the highway when suddenly 5 pedestrians appear ahead. The car's brakes happen to fail at this moment. The way to save the 5 pedestrians is to swerve the car, but doing so will cause it to hit the guardrail, leading to either the death of the passenger or one pedestrian.

In the scenario of the autonomous driving accident, driving forward will result in the death of five pedestrians, while swerve will result in the death of one pedestrian or the passenger (car owner).

If you were a designer, would you consider adopting a "driver-oriented" strategy, directly sacrificing pedestrians to protect the passenger (car owner) in the event of an accident?

Yes No

In this scenario, what is your level of moral acceptance of the "driver-oriented" approach?

(1) Totally acceptable

(2) Acceptable

(3) Somewhat acceptable

(4) Neutral

(5) Somewhat unacceptable

(6) Unacceptable

(7) Totally unacceptable

If such a "driver-oriented" autonomous car was introduced to the market, would you consider buying it?

Yes No

*Scenario of Hybrid method*

In the scenario of an autonomous driving accident, an autonomous car carrying one passenger (car owner) is speeding down the highway when suddenly 5 pedestrians appear ahead. The car's brakes happen to fail at this moment. The way to save the 5 pedestrians is to swerve the car, but doing so will cause it to hit the guardrail, leading to either the death of the passenger or one pedestrian.

In the scenario of the autonomous driving accident, driving forward will result in the death of five pedestrians, while swerve will result in the death of one pedestrian or the passenger (car owner).

If you were a designer, would you consider adopting a combined strategy of "driver- oriented" and "sacrifice the few to save the many"? This combined strategy means that in scenarios involving the passenger (car owner) and pedestrians, priority is given to protecting the passenger (car owner), while in pedestrian scenarios, a choice is made to protect the majority of pedestrians by swerve the vehicle.

Yes No

In this scenario, what is your level of moral acceptance of the combined strategy of "driver- oriented" and "sacrifice the few to save the many"?

(1) Totally acceptable

(2) Acceptable

(3) Somewhat acceptable

(4) Neutral

(5) Somewhat unacceptable

(6) Unacceptable

(7) Totally unacceptable

If such a "driver- oriented" and "sacrifice the few to save the many" autonomous car was introduced to the market, would you consider buying it?

Yes No

If the following four types of autonomous vehicles are on sale at the same time, all at the same price, which one would you buy?

An autonomous car adopting the "sacrifice the few to save the many" strategy

An autonomous car adopting the "minimize maximum fatality rate" strategy

An autonomous car adopting the "driver-oriented" strategy

An autonomous car adopting a combined "driver-oriented" and "sacrifice the few to save the many" strategy

I have other answers
